# A toxicological assessment of *Hericium erinaceus* (Lion’s mane) and *Trametes versicolor* (Turkey tail) mushroom powders

**DOI:** 10.3389/ftox.2025.1651442

**Published:** 2025-10-28

**Authors:** Kritika Mahadevan, Julie Daoust, Thomas Brendler, Ankit Chaudhary, Alimuddin Saifi, Vipin Kumar Garg

**Affiliations:** ^1^ M2 Ingredients, Vista, CA, United States; ^2^ Pharmacognosy Institute, Retzky College of Pharmacy, University of Illinois Chicago, Chicago, IL, United States; ^3^ Department of Botany and Plant Biotechnology, University of Johannesburg, Johannesburg, South Africa; ^4^ Department of Pharmaceutical Technology, Meerut Institute of Engineering and Technology (MIET), Meerut, India

**Keywords:** *Hericium erinaceus*, Lion’s mane, *Trametes versicolor*, Turkey tail, mushroom, mycelium, fruiting body, toxicity

## Abstract

*Hericium erinaceus* (Lion’s mane) and *Trametes versicolor* (Turkey Tail) mushrooms have an extensive history of use in traditional medicine and as food. Oftentimes, they are available as extract preparations produced from selected life stages such as fruiting body or mycelium. Their composition may vary based on where they are grown and the conditions of post-harvest preparation. Despite their widespread traditional use and popularity, comprehensive toxicological assessments, particularly of whole mushroom powders, remain limited. This study was conducted to evaluate whether the commercially available Organic Lion’s Mane M2-102-10 powder (*H. erinaceus* mycelial biomass and fruiting body cultured on oats) and Organic Turkey Tail M2-101-03 powder (*Trametes versicolor* mycelial biomass and primordia cultured on oats) cause acute toxicity, subchronic toxicity, and genotoxicity in rats. The tests were carried out in accordance with OECD guidelines. The results demonstrated that both Organic Lion’s Mane M2-102-10 powder and Organic Turkey Tail M2-101-03 powder did not induce acute toxicity, showed no evidence of subchronic oral toxicity in rats at doses up to 2000 mg/kg body weight/day, and exhibited no genotoxicity in either *in vitro* or *in vivo* assays.

## 1 Introduction

Mushrooms belonging to the *Hericium* and *Trametes* genera have been consumed both as food and as traditional medicinal agents due to their nutritional and bioactive benefits.

*Hericium erinaceus* (Bull.) Persoon (syn. *Hydnum erinaceus* Bull., commonly called Lion’s Mane) is a wood-decaying basidiomycete that can grow on many tree species including birch, oak, maple, and beech. The fruiting body is white when fresh and yellowish with age. It consists of a rounded solid mass of spines that are 1–4 cm long which hang down in a beardlike fashion. The mushroom is attached to the tree by a tough, thick, root-like structure (Khan et al., 2013; Thongbai et al., 2015).

Lion’s Mane is a popular culinary mushroom species that is wild-harvested and cultivated in many countries (Gry and Andersson, 2014; Tanaka, 1976). It is one of five well-known *Hericium* species occurring in North America (Arora, 1986; Christensen, 1943; McKenny and Stuntz, 1971). Among the Kashaya Pomo, a Native American people of northern California, Lion’s Mane was valued as a vegetable, typically baked or fried (Moerman, 1998). Widespread consumption of this mushroom for food is also reported from China (Hu, 2005), as well as from various countries across Asia and Europe (Usher, 1974; Živković et al., 2021). Common methods of preparation include parboiling or blanching, then sauteing or frying in butter or adding chopped to sauces and gravies (Anon, 1969; Facciola, 1990).

Lion’s Mane has been reported to have immunomodulatory, neurotrophic, neuroprotective, antioxidant, anti-tumor, anti-inflammatory, prebiotic, and gut-health promoting effects (Gravina et al., 2023; Khan et al., 2013; Szućko-Kociuba et al., 2023). The principal components of medical interest include polysaccharides, glycoproteins, and a number of diterpenes such as the hericenones, erinacines, and hericerin (Friedman, 2015; Zeng et al., 2018).

*Trametes versicolor* commonly known as Turkey Tail, is a common wood-decaying basidiomycete that grows on deadwood and dying trees across the world. It is a polypore mushroom where spores are produced on basidia inside of tubes located on the underside of the fruiting body. The thin fruiting bodies which lack stipes are highly variable in color with sharply contrasting concentric zones of color on the surface of the cap which is finely fuzzy or velvety (Camp and Ball, 2022; Zmitrovich et al., 2012).

While Turkey Tail is very common throughout the world and considered “edible” (Boa, 2004), it is not commonly consumed as food due to its tough and leathery texture. It is, however, prepared for consumption in many ways, such as boiling for teas or used for soup stocks (Arora, 1986; Hobbs, 2003). Other closely related *Polyporus* species are consumed for food (Usher, 1974). Use among North American people has been reported as winter and famine food, but also as a delicacy (sic!) (Castetter, 2025).

Turkey Tail has been associated with antitumor, immunomodulatory, antioxidant, anti-inflammatory, hypolipidemic, hepatoprotective, and antimicrobial effects (Ajibola et al., 2024; Cruz et al., 2016; Habtemariam, 2020; Darshan et al., 2024). The principal components of medical interest include polysaccharides, polysaccharopeptides, sterols, and phenolic compounds (Sun et al., 2014).

Dried and powdered preparations of the mycelia, the fruiting bodies of Lion’s Mane and Turkey Tail powders (containing mycelium and fruiting body) showed prebiotic effects, by enhancing the production of short chain fatty acids and beneficial bacteria in an *in vitro* colonic simulation study (Darshan et al., 2024; Menon et al., 2025; Daoust et al., 2025). Wildcrated sources of these species are available but are highly variable in their quality and consistency (Lu et al., 2020). Preparations made from liquid fermentation or solid-state fermentation processes provides a means to highly efficient, consistent, year-round production and allow for better quality control of the final product (Berovic and Zhong, 2023; Junior Letti et al., 2018).

Both *H. erinaceus* and *T. versicolor* have long histories of use in traditional medicine, but there are a few studies published on their potential toxicity. Most published studies on these species predominantly report on extract preparations, some of which are enriched in bioactive compounds (Chen et al., 2022; Lai et al., 2011; Lakshmanan et al., 2016; Li et al., 2014a; Li et al., 2014b). Toxicity testing to support the safety of powders produced from *H. erinaceus* mycelial biomass and fruiting body (Organic Lion’s Mane M2-102-10) and *T. versicolor* mycelial biomass and primordia (Organic Turkey Tail M2-101-03), cultured on organic oats was conducted. The two powders were evaluated in this study to check if they cause acute toxicity, sub-chronic toxicity, and genotoxicity in rats.

## 2 Pre-clinical study materials and methods

Organic Lion’s Mane M2-102-10 powder (trademarked as *M2 Lion’s Mane 102™*) and Organic Turkey Tail M2-101-03 powder produced by M2 Ingredients were investigated for their ability to induce acute toxicity, subchronic toxicity, and genotoxicity. Both substances were studied in independent toxicological studies that were carried out at the Meerut Institute of Engineering and Technology (MIET) using standardized methodology aligned with OECD guidelines.

The methodology applied in this study set closely follows that described by Chrysostomou et al. (2024), as both the investigations were carried out by the same research team within a similar timeframe. The primary distinction between the studies lies in the specific mushroom species evaluated. The research described in both the previously published study (Chrysostomou et al., 2024) and the present manuscript was funded by M2 Ingredients, who also participated in the preparation of both manuscripts.

All experimental procedures adhered to MIET’s standard operating procedures, following the institution’s internal “*Guide for the Care and Use of Experimental Animals”*. The studies were carried out in compliance with Good Laboratory Practice (GLP) standards.

### 2.1 Test material

Organic Lion’s Mane M2-102-10 powder (M2 Lion’s Mane 102™) consists of *H. erinaceus* mycelial biomass and fruiting body cultured on whole certified organic oats (*Avena sativa).* The biomass was gently dried to help break down the fungal cell walls and subsequently powdered using an industrial scale mill. Organic Turkey Tail M2-101-03 powder comprises of *T. versicolor* mycelial biomass and primordia cultured on organic whole oats. The cultured material was similarly dried and milled into a powder. The identities of the species were validated using DNA analysis of master cell cultures, along with taxonomic verification and visual examination of morphological characteristics and growth parameters throughout the cultivation cycle.

### 2.2 Acute oral toxicity

The acute oral toxicity studies were carried out in accordance with the Organization for Economic Cooperation and Development (OECD) Guidelines for the Testing of Chemicals, Test Guideline No. 425 (OECD, 2022).

Five female Wistar rats received Organic Lion’s Mane M2-102-10 powder or Organic Turkey Tail M2-101-03 powder mixed in purified water, as a single oral gavage dose at 2000 mg/kg body weight (bw). Animals were monitored for a period of 14 days post-dosing for signs of toxicity and mortality.

Additionally, groups of three female rats per dose level received single oral gavage doses of either Organic Lion’s Mane M2-102-10 powder or Organic Turkey Tail M2-101-03 powder at concentrations of 61, 195, or 625 mg/kg bw. These animals were also monitored for 14 days.

All animals underwent a 5-day acclimation period prior to dosing. Throughout the observation period, animals were assessed for clinical signs, including evaluations of muscle activity (locomotion, coordination, catatonia, tremors, and convulsions), reflex activity (visual place response, writhing response, tail pinch response, and piloerection), and secretory responses (lacrimation, salivation, sniffing, and defecation). Respiratory rate and heart rate were also monitored.

Motor activity and grip strength assessments were conducted, and body weights were recorded on Day 0 (before dosing) and on Days 1, 7, and 14. The amount of food consumed was recorded daily on a per animal cage basis.

At the conclusion of the 14-day observation period, surviving animals were euthanized using pentobarbital, followed by gross necropsy and macroscopic pathological examination.

### 2.3 90-day oral toxicity

The 90-day oral toxicity studies were conducted in accordance with the OECD Guidelines for Testing of Chemicals, No. 408 (OECD, 2018). Wistar rats (10 animals per sex per group) were assigned to treatment groups and received the test substance suspended in purified water via oral gavage at daily dose levels of 0, 500, 1,000, or 2,000 mg/kg body weight for a duration of 90 days. Before dosing, animals were fasted overnight and remained without food for an additional 2–4 h following administration. Drinking water was available without restriction, and animals were provided with food each day. All animals underwent a 5-day acclimation period prior to the initiation of dosing. Environmental conditions were maintained with 10–15 air exchanges per hour, at a temperature range of 22 °C ± 3 °C, and relative humidity maintained between 40% and 60%. A 12-h light/dark cycle was established using artificial fluorescent lighting. Animals in all groups were monitored daily for clinical signs, morbidity, and mortality. Body weights were recorded on Day 0 (prior to the first treatment dose) and subsequently, at 12-day intervals throughout the study.

At the conclusion of the 90-day treatment period, comprehensive clinical pathology assessments were conducted on all study animals. Hematological parameters included measurement of hemoglobin concentration, red and white blood cell counts, platelet count, packed cell volume, mean corpuscular volume (MCV), mean corpuscular hemoglobin (MCH), mean corpuscular hemoglobin concentration (MCHC), and differential leukocyte counts (neutrophils, eosinophils, lymphocytes, and monocytes). Clinical biochemistry assessments included total protein, albumin, total cholesterol, triglycerides, creatinine, glucose, uric acid, blood urea nitrogen (BUN), total and direct bilirubin, and enzyme activities such as alanine aminotransferase (ALT), aspartate aminotransferase (AST), and alkaline phosphatase (ALP). Thyroid hormone levels were also determined. Coagulation parameters were assessed via determination of clotting time. Urinalysis (color, clarity, pH, protein, glucose, bilirubin, and microscopic examination) was performed on all animals during the final week of the study before termination (Day 90). Food and water were temporarily withheld during the collection of blood and urine samples.

At study termination, necropsies were carried out on all animals. Absolute organ weights were recorded for the adrenals, brain, kidneys, lungs, heart, spleen, testes, uterus, and ovaries. Relative organ weights were calculated by dividing the absolute organ weight by the final body weight of each animal and expressing the result as a percentage. Histopathological assessments were subsequently carried out on tissues collected from the control and high dose group animals (FAO/WHO, 2011). Organs examined comprised the liver, kidneys, lungs, heart, brain, stomach, spleen, testes (with seminiferous tubules), uterus (including the cervix), and ovaries. In addition, lungs from animals belonging to the low- and mid-dose groups were examined histologically for potential signs of infection.

### 2.4 Bacterial reverse mutation assay

The bacterial reverse mutation assay was conducted according to OECD Guidelines for Testing of Chemicals, No. 471 (OECD, 2020) using *Salmonella typhimurium* strains TA98, TA100, TA1535, and TA, and *Escherichia coli* strain WP2 uvrA in the presence and absence of exogenous metabolic activation system (S9 fraction liver extract). Each test material was evaluated in a preliminary cytotoxicity screen followed by a definitive assay and a confirmatory repeat assay at concentrations up to 5,000 µg/plate. A co-factor–supplemented S9 fraction, prepared from Arcolor™ 1254-induced rat liver, was used as the metabolic activation system at concentrations of 5% for the definitive assay and 10% for the repeat assay. Dimethyl sulfoxide (DMSO) served as the solvent for the test items. Each assay was conducted in triplicate, and revertant colonies were enumerated using an automated digital colony counter. For each concentration, the mean number of revertant colonies and the corresponding standard deviation were calculated from the triplicate plates.

### 2.5 *In vivo* micronucleus test

An *in vivo* micronucleus test was carried out to assess the clastogenic and/or aneugenic potential of Organic Lion’s Mane M2-102-10 powder and Organic Turkey Tail M2-101-03 powder. The study followed OECD Test Guideline No. 474 (OECD, 2014). The test materials were suspended in purified water prior to administration, with phosphate-buffered saline serving as the vehicle control. Cyclophosphamide (50 mg/kg bw), a known genotoxic agent, was used as positive control. Swiss albino mice (five males and five females per group), were used in each study. Test groups received oral gavage doses of 0 (control), 300, 1,000, or 2,000 mg/kg bw of either Organic Lion’s Mane M2-102-10 powder or Organic Turkey Tail M2-101-03 powder or the positive control. A satellite group also received 2,000 mg/kg bw to assess potential delayed effects. Dosing was conducted twice at 14-h intervals. The animals in the treatment, vehicle, and positive control groups were euthanized 24 h after the final dose. Animals from the satellite group were euthanized after 48 h post final administration. Bone marrow samples were collected and processed for micronucleus analysis, including staining, fixation, and microscopic examination at ×100 magnification. For each animal, micronucleated polychromatic erythrocytes (PCEs) were counted from 2,000 cells. Additionally, normochromatic erythrocytes (NCEs) were counted in a sample of 1,000 erythrocytes to determine the frequency of micronucleated NCEs. The ratio of PCE to NCE was calculated, and mean values with standard deviations were determined for each group.

### 2.6 Statistical analysis

For the 90-day oral toxicity study, statistical analyses were performed using Microsoft Excel. Mean values and standard deviations were calculated separately for males and females within each dosage group for all test parameters, including hematology, clinical chemistry, body weight, and organ weights. Statistical significance (p < 0.05) between each treatment group against their respective control group was conducted using independent T-Test for males and females. For the *in vivo* micronucleus test, statistical comparisons among dose groups were performed using one-way ANOVA.

## 3 Results

### 3.1 Pre-clinical study results

#### 3.1.1 Acute oral toxicity

Acute oral toxicity testing conducted in Wistar rats revealed no mortality in any group within the 14-day observation period following the receipt of a dose of up to 2000 mg/kg bw of either Organic Lion’s Mane M2-102-10 powder or Organic Turkey Tail M2-101-03 powder. There were no treatment-related clinical signs, body weight alterations, or macroscopic abnormalities detected. These findings indicate that both test substances are non-lethal at the limit dose of 2000 mg/kg bw. Accordingly, the median lethal dose (LD_50_) for each test item was estimated to be greater than 2000 mg/kg bw. Tables 1, 2 summarize the findings from acute oral toxicity studies of Organic Lion’s Mane M2-102-10 and Organic Turkey Tail M2-101-03 powders.

**TABLE 1:** Summary results of the acute oral toxicity study with Organic Lion’s Mane M2-102-10 powder.

Dose (mg/kg bw/day)		61	195	625	2000
Mortality observed		No	No	No	No
Body weight (g)	Day 0	196	194	193	192
	Day 1	196	195	194	193
	Day 7	199	199	197	195
	Day 14	202	200	199	196
Macroscopic Findings (Abnormalities detected)		None	None	None	None

**TABLE 2:** Summary results of the acute oral toxicity study with Organic Turkey Tail M2-101-03 powder.

Dose (mg/kg bw/day)		61	195	625	2000
Mortality observed		No	No	No	No
Body weight (g)	Day 0	186	183	192	191
	Day 1	187	184	193	191
	Day 7	188	185	193	193
	Day 14	192	187	196	196
Macroscopic Findings (Abnormalities detected)		None	None	None	None

#### 3.1.2 90-day oral toxicity

##### 3.1.2.1 Organic Lion’s mane M2-102-10 powder

**FIGURE 1:**
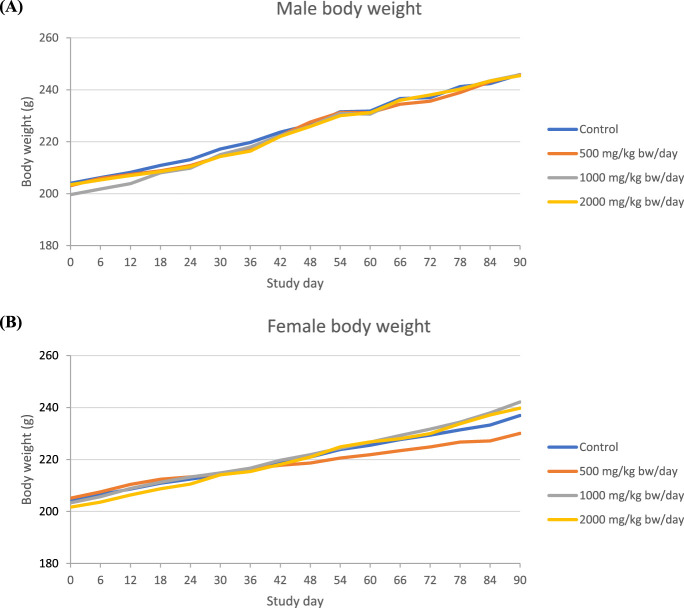
Body weight in 90-day repeated dose toxicity study with Organic Lion’s Mane M2-102-10 powder (**(A)**=Male, **(B)**: Female).

In the 90-day study of Organic Lion’s Mane M2-102-10 powder, there were no statistically significant or treatment-related effects observed in both sexes of rats at any dose level with respect to mortality, clinical observations, ophthalmologic examinations, urinalysis results, or gross and microscopic pathology findings. Additionally, the body weight of male and female rats showed no significant alterations in any treatment group when compared to their respective control groups (Figure 1).

Changes in the mass of organs in the treatment groups are summarized in Table 3. The mass of adrenal glands in the high dose female rats were significantly higher in comparison to the control group (p < 0.05). At the 1,000 mg/kg bw/day dose level, both absolute and relative spleen weights were significantly elevated in both sexes (p < 0.05). Similarly, liver weights (absolute and relative) were significantly higher in males and females at the higher dose levels (p < 0.05). In contrast, heart weights were significantly lower in both sexes at the 2000 mg/kg bw/day dose level relative to controls (p < 0.05). The mass of ovaries (absolute and relative) was also significantly elevated in females receiving high doses of the powder (p < 0.05). Despite these changes, no corresponding microscopic abnormalities were identified during histopathological examination of any organs, including the adrenal glands, spleen, heart, and ovaries, in the high-dose group.

**TABLE 3:** Organ weights in the 90-day repeated dose study with Organic Lion’s Mane M2-102-10 powder.

Dose group (mg/kg bw/day)	Male				Female			
	Control	500	1,000	2000	Control	500	1,000	2000
Absolute organ weight								
Adrenals (g)	0.60 ± 0.04	0.60 ± 0.02	0.59 ± 0.01	0.61 ± 0.01	0.57 ± 0.04	0.60 ± 0.01	0.59 ± 0.03	0.64 ± 0.02*
Liver (g)	8.05 ± 0.04	8.45 ± 0.30*	8.43 ± 0.57	8.05 ± 0.05	8.09 ± 0.13	8.63 ± 0.29*	8.85 ± 0.56*	8.06 ± 0.04
Kidneys (g)	1.79 ± 0.04	1.79 ± 0.05	1.78 ± 0.05	1.80 ± 0.05	1.83 ± 0.05	1.78 ± 0.06	1.79 ± 0.05	1.81 ± 0.06
Brain (g)	2.29 ± 0.24	2.12 ± 0.05*	2.20 ± 0.09	2.15 ± 0.02	2.24 ± 0.16	2.16 ± 0.07	2.12 ± 0.06*	2.18 ± 0.05
Heart (g)	0.86 ± 0.03	0.84 ± 0.03	0.85 ± 0.03	0.79 ± 0.05*	0.88 ± 0.05	0.83 ± 0.03*	0.86 ± 0.04	0.82 ± 0.05*
Spleen (g)	0.82 ± 0.04	0.80 ± 0.03	0.86 ± 0.04*	0.82 ± 0.04	0.82 ± 0.04	0.80 ± 0.05	0.91 ± 0.05*	0.79 ± 0.05
Lungs (g)	1.92 ± 0.04	1.90 ± 0.05	1.88 ± 0.04*	1.92 ± 0.05	1.90 ± 0.05	1.88 ± 0.05	1.87 ± 0.03	1.88 ± 0.05
Testes (g)	2.62 ± 0.10	2.63 ± 0.17*	2.47 ± 0.11*	2.66 ± 0.18				
Ovaries (g)					0.04 ± 0.02	0.07 ± 0.04	0.09 ± 0.03*	0.10 ± 0.11
Relative to body weight (%)								
Adrenals (%)	0.2 ± 0.01	0.2 ± 0.0	0.2 ± 0.0	0.2 ± 0.00	0.2 ± 0.0	0.3 ± 0.0*	0.2 ± 0.0	0.3 ± 0.0*
Liver (%)	3.3 ± 0.01	3.4 ± 0.1*	3.4 ± 0.2*	3.3 ± 0.02	3.4 ± 0.1	3.8 ± 0.1*	3.7 ± 0.2*	3.4 ± 0.0*
Kidneys (%)	0.7 ± 0.02	0.7 ± 0.0	0.7 ± 0.0	0.7 ± 0.02	0.8 ± 0.0	0.8 ± 0.0	0.7 ± 0.0*	0.8 ± 0.0
Brain (%)	0.9 ± 0.10	0.9 ± 0.0*	0.9 ± 0.0	0.9 ± 0.01	0.9 ± 0.1	0.9 ± 0.0	0.9 ± 0.0*	0.9 ± 0.0
Heart (%)	0.3 ± 0.01	0.3 ± 0.0	0.3 ± 0.0	0.3 ± 0.02*	0.4 ± 0.0	0.4 ± 0.0	0.4 ± 0.0	0.3 ± 0.0*
Spleen (%)	0.3 ± 0.02	0.3 ± 0.0	0.4 ± 0.0*	0.3 ± 0.02	0.3 ± 0.0	0.3 ± 0.0	0.4 ± 0.0*	0.3 ± 0.0
Lungs (%)	0.8 ± 0.02	0.8 ± 0.0	0.8 ± 0.0*	0.8 ± 0.02	0.8 ± 0.0	0.8 ± 0.0	0.8 ± 0.0*	0.8 ± 0.0
Testes (%)	1.1 ± 0.04	1.1 ± 0.0	1.0 ± 0.0*	1.1 ± 0.08				
Ovary (%)					0.0 ± 0.0	0.03 ± 0.02	0.04 ± 0.0*	0.04 ± 0.05

Data shown as mean ± SD.

*Statistical significance compared to control, p < 0.05.

N = 10 animals/sex/group.

Some values appear to be zero (0.0 or 0.00) due to rounding.

Clinical chemistry parameters (Table 4) did not show any biologically meaningful adverse effects in either sexes of rats. A few parameters showed a statistically significant difference between control and treatment groups. Total bilirubin was significantly elevated in female rats at higher doses in comparison to the control group. Alkaline phosphatase showed a small dip only in female rats, and only at the 1,000 mg/kg bw/day dose. Statistically significant (p < 0.05) elevation in aspartate aminotransferase (AST) levels was noted in the 500 mg/kg bw/day dose group, followed by a significant decline at higher dose levels in both male and female rats, relative to the control group. Triglycerides were elevated significantly (p < 0.05) in the 2000 mg/kg bw/day treatment group, whereas total cholesterol was lower (but not linear in trend) in higher treatment doses in both the sexes of rats. Glucose level was significantly increased in males and females in all treatment dosages relative to controls (p < 0.05). TSH level showed a slight increase in the 2000 mg/kg bw/day treated male rats relative to the control group that was statistically significant (p < 0.05).

**TABLE 4:** Clinical chemistry parameters in the 90-day repeated dose study with Organic Lion’s Mane M2-102-10 powder.

Dose group (mg/kg bw/day)	Male				Female			
	Control	500	1,000	2000	Control	500	1,000	2000
Albumin (g/dL)	3.9 ± 0.4	4.0 ± 0.4	3.6 ± 0.3	3.7 ± 0.3	3.9 ± 0.4	3.8 ± 0.5	3.7 ± 0.4	4.0 ± 0.5
Total Protein (g/dL)	6.9 ± 0.3	6.8 ± 0.3	6.8 ± 0.3	6.8 ± 0.3	7.0 ± 0.4	6.8 ± 0.2	6.8 ± 0.3	6.8 ± 0.3
Bilirubin Total (mg/dL)	0.5 ± 0.1	0.5 ± 0.1	0.5 ± 0.1	0.5 ± 0.1	0.4 ± 0.1	0.6 ± 0.1*	0.5 ± 0.1	0.6 ± 0.1*
Bilirubin Direct (mg/dL)	0.2 ± 0.1	0.2 ± 0.0	0.2 ± 0.0	0.2 ± 0.0	0.2 ± 0.0	0.2 ± 0.0*	0.2 ± 0.0	0.2 ± 0.0
Alanine Amino Transferase (IU/L)	42.6 ± 7.3	45.4 ± 5.8	38.2 ± 6.1	45.3 ± 5.0	44.3 ± 7.2	44.0 ± 5.4	42.2 ± 8.9	45.4 ± 6.3
Alkaline phosphatase (IU/L)	140 ± 0.6	140 ± 1.1	140 ± 1.5	140 ± 0.6	140 ± 0.5	139 ± 1.2	139 ± 1.2*	140 ± 0.8
Aspartate Amino Transferase (IU/L)	70.6 ± 4.1	87.9 ± 6.0*	53.0 ± 6.5*	57.4 ± 5.3*	66.5 ± 7.8	84.8 ± 5.6*	56.3 ± 7.5*	56.9 ± 4.2*
Blood Urea Nitrogen (mg/dL)	17.1 ± 4.1	20.6 ± 3.5	15.7 ± 3.2	17.9 ± 4.2	18.9 ± 2.9	17.5 ± 4.5	16.5 ± 3.0	19.0 ± 4.5
Uric acid	1.2 ± 0.4	1.1 ± 0.4	1.1 ± 0.3	1.2 ± 0.4	1.1 ± 0.3	1.1 ± 0.4	1.2 ± 0.4	1.2 ± 0.4
Creatinine (mg/dL)	0.4 ± 0.1	0.4 ± 0.1	0.5 ± 0.1	0.4 ± 0.1	0.4 ± 0.1	0.6 ± 0.1	0.4 ± 0.1	0.5 ± 0.2
Total Cholesterol (mg/dL)	78.0 ± 5.3	57.8 ± 6.8*	47.5 ± 3.8*	60.0 ± 5.2*	77.0 ± 6.1	61.7 ± 6.3*	46.9 ± 3.0*	61.6 ± 4.3*
Glucose (mg/dL)	189 ± 5.1	201 ± 5.3*	195 ± 6.1*	204 ± 2.6*	189 ± 6.9	198 ± 6.2*	198 ± 6.1*	204 ± 2.3*
Triglycerides (mg/dL)	46.9 ± 6.3	45.8 ± 7.9	50.6 ± 2.8	56.9 ± 6.2*	46.9 ± 5.1	45.8 ± 6.8	49.2 ± 2.4	54.9 ± 5.7*
T3 (nmol/L)	1.3 ± 0.0	1.4 ± 0.1	1.3 ± 0.1	1.3 ± 0.1	1.3 ± 0.1	1.4 ± 0.1	1.3 ± 0.1	1.4 ± 0.1
T4 (nmol/L)	2.4 ± 0.2	2.6 ± 0.2	2.5 ± 0.2	2.5 ± 0.2	2.4 ± 0.2	2.5 ± 0.3	2.4 ± 0.2	2.5 ± 0.2
TSH (ng/mL)	1.4 ± 0.1	1.3 ± 0.1	1.3 ± 0.1*	1.6 ± 0.1*	1.3 ± 0.1	1.3 ± 0.2	1.3 ± 0.1	1.4 ± 0.1

Data shown as mean ± SD.

*Statistical significance compared to control, p < 0.05.

N = 10 animals/sex/group.

Some values appear to be zero (0.0) due to rounding.

Results from the hematological parameters (Table 5) indicated a statistically significant (p < 0.05) reduction in hemoglobin in the 2000 mg/kg bw/day dose group and a reduction in total red cell count in the 1,000 mg/kg bw/day dose group in both male and female rats compared to the controls. A decrease in platelet count at the 500 mg/kg bw/day dose in both sexes of rats, as well as in female rats at the 2000 mg/kg bw/day dose was noted, when compared with controls. Conversely, an increase in monocyte count was observed in the 2000 mg/kg bw/day dose group in both the male and female rats. Mean corpuscular hemoglobin was lower in male and female rats at the 500 mg/kg bw/day dose, and at 1,000 mg/kg bw/day dose in male rats compared to the controls. The Mean corpuscular hemoglobin concentration (MCHC) was significantly lower in the 2000 mg/kg bw/day dose group in male rats (p < 0.05).

**TABLE 5:** Hematology results in the 90-day repeated dose study with Organic Lion’s Mane M2-102-10 powder.

Dose group (mg/kg bw/day)	Male				Female			
	Control	500	1,000	2000	Control	500	1,000	2000
Hemoglobin (Hb) (g/dL)	11.4 ± 0.8	11.7 ± 1.1	12.2 ± 0.6*	10.4 ± 0.9*	12 ± 0.8	12 ± 0.7	11.7 ± 0.6	10.2 ± 0.7*
Total red cell count (x10^6^/µl)	8.0 ± 0.5	7.8 ± 0.5	7.6 ± 0.2*	7.6 ± 0.5	8.0 ± 0.5	8.1 ± 0.5	7.6 ± 0.4*	7.9 ± 0.4
Total white cell count (Total WBC) (x10^3^/µl)	6.7 ± 1.0	6.9 ± 0.7	7 ± 1.0	6.4 ± 0.9	6.2 ± 0.8	6.6 ± 0.9	6.2 ± 0.7	6.1 ± 0.8
Packed cell volume (PCV) (%)	40 ± 1.2	41 ± 0.9	41 ± 1.4	39 ± 1.3	39 ± 0.9	40 ± 1.3	41 ± 1.4*	39 ± 1.6
Platelet Count, (x10^5^/cmm)	794 ± 4.6	769 ± 4.7*	794 ± 2.6	790 ± 5.6	794 ± 4.1	772 ± 3.5*	792 ± 3.6	785 ± 10*
Mean corpuscular volume (MCV) (fL)	59 ± 2.8	61 ± 4.4	59 ± 3.7	61 ± 2.1	61 ± 3.0	63 ± 2.6	60 ± 2.9	62 ± 1.7
Mean corpuscular hemoglobin (MCH) (pg)	16 ± 0.5	15 ± 0.6*	15 ± 1.0*	16 ± 0.7	16 ± 0.6	15 ± 0.5*	15 ± 1.2	16 ± 0.9
Mean corpuscular hemoglobin concentration (MCHC) (g/dL)	29 ± 1.2	29 ± 1.1	29 ± 1.8	28 ± 1.1*	29 ± 1.4	29 ± 1.2	30 ± 1.8	28 ± 1.2
Lymphocyte (%)	66 ± 1	66 ± 0.8	66 ± 1.0	66 ± 1.0	67 ± 0.9	67 ± 1.0	66 ± 1.0	66 ± 0.7
Neutrophils (%)	29.9 ± 3.9	30 ± 2.5	31 ± 3	31 ± 2.3	31.2 ± 2.5	30 ± 3.1	31 ± 3.4	32 ± 1.9
Monocytes (%)	1.9 ± 0.7	1.8 ± 0.7	1.9 ± 0.5	2.8 ± 0.5*	2.1 ± 0.7	1.9 ± 0.6	1.7 ± 0.7	3.3 ± 0.8*
Eosinophils (%)	2.3 ± 0.7	2.3 ± 0.9	2.2 ± 0.5	2.2 ± 0.8	2.8 ± 0.6	2.8 ± 1.0	2.4 ± 0.6	2.7 ± 0.6

Data shown as mean ± SD.

*Statistical significance compared to control, p < 0.05.

N = 10 animals/sex/group.

Some values appear to be zero (0.0) due to rounding.

The identified findings and alterations observed lacked a dose-dependent trend, remained within the range of normal biological variation, and were not regarded to be adverse effects. Accordingly, the “no observed adverse effect level” (NOAEL) for Organic Lion’s Mane M2-102-10 powder was determined to be 2000 mg/kg body weight/day based on the absence of adverse effects at the highest dosage studied.

##### 3.1.2.2 Organic Turkey tail M2-101-03 powder

**FIGURE 2:**
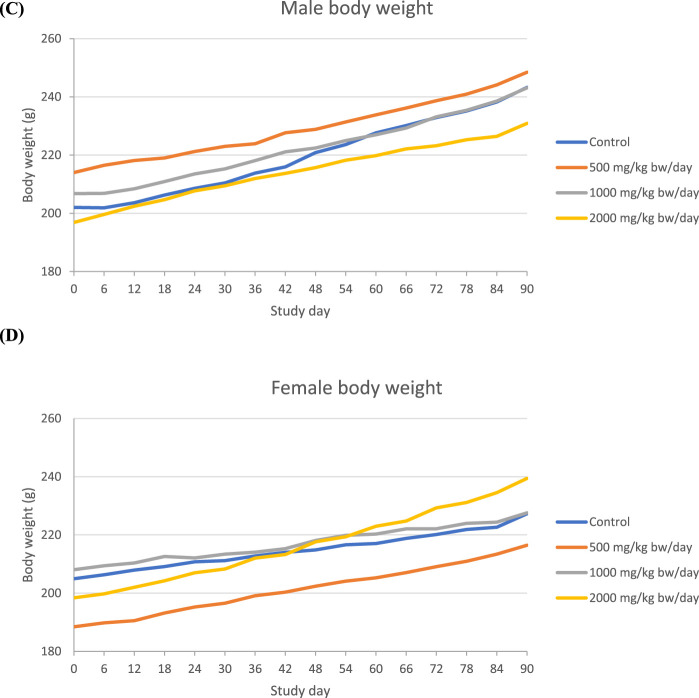
Body weight in 90-day repeated dose toxicity study with Organic Turkey Tail M2-101-03 powder (**(A)**=Male, **(B)**: Female).

In the 90-day study of Organic Turkey Tail M2-101-03 powder, no statistically significant or treatment-related effects were observed in male or female rats at any dose level with respect to mortality, clinical observations, ophthalmologic examinations, urinalysis results, or gross and microscopic pathology findings. A small decrease in the body weight of male rats and a small increase in body weight of the female rats that were on 2000 mg/kg bw/day dose was observed compared to their respective control groups (Figure 2).

Table 6 summarizes the organ weight findings from the 90-day oral toxicity study of Organic Turkey Tail M2-101-03 powder. At the 2000 mg/kg bw/day dose, both male and female rats showed statistically significant increases (p < 0.05) in adrenal and brain weights in comparison to control rats. Kidney weights (absolute and relative to body weight) were slightly but significantly decreased (p < 0.05) in both sexes at the higher dose levels. Additionally, in high-dose groups, the absolute heart and spleen weights were significantly elevated in both sexes (p < 0.05), while male lung weights showed a small but statistically significant reduction. Testes weights (absolute and relative to body weight) at 2000 mg/kg bw/day dose were significantly increased compared with controls in males (p < 0.05).

**TABLE 6:** Organ weights in the 90-day repeated dose study with Organic Turkey Tail M2-101-03 powder.

Dose group (mg/kg bw/day)	Male				Female			
	Control	500	1,000	2000	Control	500	1,000	2000
Absolute organ weight								
Adrenals (g)	0.58 ± 0.02	0.60 ± 0.01*	0.60 ± 0.01	0.64 ± 0.01*	0.58 ± 0.04	0.60 ± 0.01	0.60 ± 0.01	0.64 ± 0.01*
Liver (g)	8.39 ± 0.22	8.45 ± 0.03	8.52 ± 0.11	8.45 ± 0.03	8.44 ± 0.17	8.41 ± 0.05	8.52 ± 0.29	8.46 ± 0.03
Kidneys (g)	1.83 ± 0.03	1.82 ± 0.03	1.87 ± 0.04*	1.63 ± 0.06*	1.82 ± 0.04	1.81 ± 0.04	1.85 ± 0.03	1.61 ± 0.06*
Brain (g)	2.18 ± 0.38	2.04 ± 0.04	2.19 ± 0.04	2.63 ± 0.03*	2.20 ± 0.33	2.06 ± 0.02	2.21 ± 0.03	2.62 ± 0.03*
Heart (g)	0.80 ± 0.04	0.82 ± 0.02	0.83 ± 0.04	0.98 ± 0.04*	0.81 ± 0.03	0.83 ± 0.03	0.84 ± 0.03	0.94 ± 0.03*
Spleen (g)	0.80 ± 0.02	0.81 ± 0.03	0.84 ± 0.02*	0.85 ± 0.03*	0.80 ± 0.02	0.80 ± 0.03	0.84 ± 0.02*	0.86 ± 0.02*
Lungs (g)	1.94 ± 0.06	1.87 ± 0.06*	1.90 ± 0.05	1.88 ± 0.03*	1.88 ± 0.05	1.91 ± 0.05	1.88 ± 0.04	1.85 ± 0.03
Testes (g)	2.44 ± 0.04	2.45 ± 0.01	2.44 ± 0.08	2.64 ± 0.02*				
Ovaries (g)					0.06 ± 0.00	0.07 ± 0.00	0.06 ± 0.00	0.08 ± 0.03
Relative to body weight (%)								
Adrenals (%)	0.2 ± 0.01	0.2 ± 0.0	0.2 ± 0.0	0.3 ± 0.0*	0.3 ± 0.0	0.3 ± 0.0	0.3 ± 0.0	0.3 ± 0.0
Liver (%)	3.4 ± 0.09	3.4 ± 0.0	3.5 ± 0.0	3.7 ± 0.0*	3.7 ± 0.1	3.9 ± 0.0*	3.7 ± 0.1	3.5 ± 0.0*
Kidneys (%)	0.8 ± 0.01	0.7 ± 0.0*	0.8 ± 0.0*	0.7 ± 0.0*	0.80 ± 0.0	0.84 ± 0.0*	0.8 ± 0.0	0.7 ± 0.0*
Brain (%)	0.9 ± 0.15	0.8 ± 0.0	0.9 ± 0.0	1.1 ± 0.0*	0.97 ± 0.1	0.95 ± 0.0	0.97 ± 0.0	1.1 ± 0.0*
Heart (%)	0.3 ± 0.02	0.3 ± 0.0	0.3 ± 0.0	0.4 ± 0.0*	0.4 ± 0.0	0.4 ± 0.01*	0.4 ± 0.0	0.4 ± 0.0*
Spleen (%)	0.3 ± 0.01	0.3 ± 0.0	0.3 ± 0.0*	0.4 ± 0.0*	0.3 ± 0.0	0.4 ± 0.0*	0.4 ± 0.0*	0.4 ± 0.0
Lungs (%)	0.8 ± 0.03	0.8 ± 0.0*	0.8 ± 0.0	0.8 ± 0.00	0.8 ± 0.0	0.9 ± 0.0*	0.8 ± 0.0	0.8 ± 0.0
Testes (%)	1.0 ± 0.02	0.98 ± 0.01*	1.00 ± 0.03	1.1 ± 0.0*				
Ovary (%)					0.03 ± 0.01	0.03 ± 0.00	0.03 ± 0.00	0.03 ± 0.0

Data shown as mean ± SD.

*Statistical significance compared to control, p < 0.05.

N = 10 animals/sex/group.

Some values appear to be zero (0.0 or 0.00) due to rounding.

Only a few clinical chemistry parameters showed significant changes (Table 7). Alanine Amino Transferase was significantly increased (p < 0.05) in female rats at higher doses compared to the control group. Triglycerides increased significantly (p < 0.05) in the 2000 mg/kg bw/day dose group in both sexes compared to the controls. The thyroid hormones (T3, T4, TSH) also showed a statistically significant increase (p < 0.05) at higher doses in males and females.

**TABLE 7:** Clinical chemistry parameters in the 90-day repeated dose study with Organic Turkey Tail M2-101-03 powder.

Dose group (mg/kg bw/day)	Male				Female			
	Control	500	1,000	2000	Control	500	1,000	2000
Albumin (g/dL)	3.2 ± 0.4	3.2 ± 0.4	3.3 ± 0.3	3.1 ± 0.1	3.1 ± 0.4	3.1 ± 0.4	3.3 ± 0.3	3.3 ± 0.4
Total Protein (g/dL)	7.2 ± 0.5	7.2 ± 0.2	7.1 ± 0.2	7.3 ± 0.4	7.4 ± 0.2	7.35 ± 0.2	7.3 ± 0.2	7.5 ± 0.2
Bilirubin Total (mg/dL)	0.3 ± 0.1	0.3 ± 0.0	0.3 ± 0.1	0.3 ± 0.1	0.4 ± 0.0	0.36 ± 0.0	0.3 ± 0.1	0.3 ± 0.1
Bilirubin Direct (mg/dL)	0.2 ± 0.0	0.2 ± 0.1	0.2 ± 0.1	0.2 ± 0.0	0.23 ± 0.1	0.23 ± 0.1	0.2 ± 0.0	0.2 ± 0.0
Alanine Amino Transferase (IU/L)	23.6 ± 7.1	23.6 ± 5.0	24.9 ± 5.3	27.5 ± 4.9	20.4 ± 5.0	20.4 ± 5.0	29.0 ± 7.8*	34.1 ± 7.2*
Alkaline phosphatase (IU/L)	142.8 ± 0.9	142.8 ± 0.5	142.6 ± 1.1	143 ± 1.4	143.3 ± 0.5	143.3 ± 0.5	143.2 ± 1.0	143.0 ± 1.3
Aspartate amino Transferase (IU/L)	63.4 ± 11	63.4 ± 5.8	58.3 ± 1.7	71.4 ± 6.9	65.3 ± 5.8	65.3 ± 5.8	57 ± 2.2*	65.9 ± 2.1
Blood Urea Nitrogen (mg/dL)	20.4 ± 2.2	20.4 ± 2.0	22.2 ± 2.3	19.7 ± 2.8	21.4 ± 2.0	21.4 ± 2.0	20.2 ± 2.5	19.9 ± 1.2
Uric acid	1.2 ± 0.5	1.2 ± 0.4	1.0 ± 0.4	0.9 ± 0.2	1.0 ± 0.4	1.0 ± 0.4	0.7 ± 0.4	0.7 ± 0.3
Creatinine (mg/dL)	0.5 ± 0.1	0.5 ± 0.1	0.6 ± 0.2	0.6 ± 0.1	0.57 ± 0.1	0.57 ± 0.1	0.5 ± 0.1	0.6 ± 0.1
Total Cholesterol (mg/dL)	54.5 ± 3.2	54.5 ± 3.8	55.6 ± 2.9	52.3 ± 1.9	53.4 ± 3.8	53.4 ± 3.8	53.9 ± 5.1	55.5 ± 6.1
Glucose (mg/dL)	155.4 ± 1.5	155.4 ± 1.3	154.8 ± 2.0	153.8 ± 2.5	155.8 ± 1.3	155.8 ± 1.3	154.4 ± 2.3	155.6 ± 2.0
Triglycerides (mg/dL)	58.6 ± 3.1	58.6 ± 2.5	58.7 ± 5.2	98.8 ± 3.5*	55.1 ± 2.5	55.1 ± 2.5	57.0 ± 4.3	101.9 ± 4.9*
T3 (nmol/L)	1.1 ± 0.0	1.1 ± 0.0	1.7 ± 0.0*	1.4 ± 0.1*	1.13 ± 0.0	1.1 ± 0.0	1.7 ± 0.0*	1.4 ± 0.1*
T4 (nmol/L)	2.4 ± 0.0	2.4 ± 0.0	2.9 ± 0.0*	2.7 ± 0.0*	2.41 ± 0.0	2.4 ± 0.0	2.9 ± 0.1*	2.7 ± 0.0*
TSH (ng/mL)	1.8 ± 0.0	1.8 ± 0.0	2.1 ± 0.1*	1.8 ± 0.4	1.8 ± 0.0	1.8 ± 0.0	2.1 ± 0.0*	2.0 ± 0.2*

Data shown as mean ± SD.

*Statistical significance compared to control, p < 0.05.

N = 10 animals/sex/group.

Some values appear to be zero (0.0) due to rounding.

Hematological parameters (Table 8) indicated a statistically significant (p < 0.05) decrease in hemoglobin, total red blood cell count, total WBC, packed cell volume in male and female rats at higher doses compared to control groups. Mean corpuscular volume (MCV) was significantly increased only in female rats at only the 1,000 mg/kg bw/day dosage in comparison to the control group. A statistically significant (p < 0.05) decrease in the mean corpuscular hemoglobin concentration (MCHC) was noted at 1,000 mg/kg bw/day and a significant increase at 2000 mg/kg bw/day in both males and females. Neutrophils and eosinophils were significantly (p < 0.05) lower at higher doses in male rats compared to the control group. A statistically significant (p < 0.05) reduction in monocytes was observed only in female rats in only the 2000 mg/kg bw/day dose group compared to the control.

**TABLE 8:** Hematology results in the 90-day repeated dose study with Organic Turkey Tail M2-101-03 powder.

Dose group (mg/kg bw/day)	Male				Female			
	Control	500	1,000	2000	Control	500	1,000	2000
Hemoglobin (Hb) (g/dL)	13.6 ± 1.4	12.6 ± 1.0	12.4 ± 1.1*	11.8 ± 0.9*	13.8 ± 1.6	12.8 ± 0.9	12.4 ± 1.4	12.0 ± 1.0*
Total red cell count (x10^6^/µl)	8.0 ± 0.3	7.8 ± 0.4	7.2 ± 0.6*	6.5 ± 0.8*	8.0 ± 0.4	7.8 ± 0.4	7.0 ± 0.5*	6.1 ± 0.5*
Total white cell count (Total WBC) (x10^3^/µl)	7.3 ± 1.0	7.1 ± 1.1	6.7 ± 0.7	6.4 ± 0.7*	8.2 ± 0.7	5.8 ± 0.8*	6.6 ± 0.8*	6.4 ± 1.0*
Packed cell volume (PCV) (%)	44 ± 3.1	44 ± 1.1	40 ± 1.1*	38 ± 1.3*	45 ± 2	44 ± 1.3	41 ± 1.2*	39 ± 0.9*
Platelet Count, (x10^5^/cmm)	734 ± 64	777 ± 57	861 ± 81*	687 ± 64	765 ± 72	816 ± 36	889 ± 38*	719 ± 33.2
Mean corpuscular volume (MCV) (fL)	56 ± 4.1	56 ± 1.4	57 ± 1.5	55 ± 1.5	54 ± 3.2	54 ± 1.4	57 ± 1.3*	56 ± 1.7
Mean corpuscular hemoglobin (MCH) (pg)	17 ± 1.0	16 ± 0.6	17 ± 0.8	17 ± 0.8	17 ± 0.8	16 ± 0.5*	17 ± 1.0	17 ± 1.5
Mean corpuscular hemoglobin concentration (MCHC) (g/dL)	29 ± 0.9	29 ± 0.9	27 ± 0.8*	31 ± 1.0*	29 ± 1.5	29 ± 1.0	26 ± 1.8*	31 ± 1.5*
Lymphocyte (%)	67 ± 2.6	68 ± 1.3	68 ± 2.0	66 ± 1.9	68 ± 1.7	69 ± 1.7	68 ± 1.5	66 ± 1.6
Neutrophils (%)	32 ± 1.7	28 ± 1.8*	28 ± 3.7*	29 ± 2.7*	28 ± 2.5	28 ± 2.6	29 ± 3.5	29 ± 3.6
Monocytes (%)	2.2 ± 0.5	2.1 ± 0.8	1.7 ± 0.6	1.8 ± 0.8	1.8 ± 0.6	1.9 ± 0.5	1.6 ± 0.4	1.4 ± 0.3*
Eosinophils (%)	3.0 ± 0.6	1.8 ± 0.6*	2.0 ± 0.6*	3 ± 0.3	2.7 ± 0.7	2.7 ± 0.4	2.8 ± 0.5	2.3 ± 0.6

Data shown as mean ± SD.

*Statistical significance compared to control, p < 0.05.

N = 10 animals/sex/group.

Some values appear to be zero (0.0) due to rounding.

The observed changes demonstrated no dose-dependent pattern and remained within the bounds of normal biological variation. No associated histopathological correlates were identified. The findings were therefore deemed to be non-adverse. Accordingly, the no observed adverse effect level (NOAEL) for Organic Turkey Tail M2-101-03 powder was established at 2000 mg/kg bw/day, which was the highest dosage tested in the study.

#### 3.1.3 Bacterial reverse mutation assay

All components of the assay were deemed valid. Based on cytotoxicity evaluations, the selected test concentrations for both Organic Lion’s Mane M2-102-10 and Organic Turkey Tail M2-101-03 powders in the definitive and independent repeat assays were 50, 100, 500, 1,000, and 5,000 μg/plate. These concentrations were tested in the presence and absence of metabolic activation using an S9 mix. Across all tested strains—*S. typhimurium* TA1535, TA97a, TA98, TA100, and *E. coli* WP2 uvrA—no treatment-related, dose-dependent, or statistically significant increases in revertant colony numbers were observed under either condition. These results were consistent across both the plate incorporation method and for each test substance (see Tables 9, 10). The results of the study indicate that Organic Lion’s Mane M2-102-10 powder and Organic Turkey Tail M2-101-03 powder did not induce gene mutations in bacterial test systems, either in the presence or absence of metabolic activation.

**TABLE 9:** Results of the bacterial reverse mutation assay with Organic Lion’s Mane M2-102-10 powder.

	Revertant colonies per plate									
Concentration (µg/plate)	*Salmonella typhimurium*								*Escherichia coli*	
	TA98		TA100		TA1535		TA97a		WP2 *uvrA*	
	-S9	+S9 (5%)	-S9	+S9 (5%)	-S9	+S9 (5%)	-S9	+S9 (5%)	-S9	+S9 (5%)
50	27.5 ± 5.2	30.2 ± 4.5	138 ± 4.3	165 ± 3.1	12.7 ± 4.5	11.1 ± 3.9	118 ± 2.5	156 ± 2.7	61.2 ± 3.5	66.0 ± 2.2
100	33.4 ± 4.3	33.1 ± 4.1	139 ± 4.6	131 ± 3.3	13.3 ± 3.53	12.4 ± 3.4	122 ± 4.1	145 ± 3.7	65.5 ± 6.6	65.2 ± 4.2
500	34.5 ± 3.5	34.7 ± 3.4	141 ± 4.5	153 ± 3.5	14.2 ± 3.5	14.2 ± 3.7	122 ± 4.6	135 ± 4.5	67.4 ± 3.3	68.5 ± 4.4
1,000	36.7 ± 4.0	37.1 ± 3.0	145 ± 4.6	156 ± 3.8	14.9 ± 4.5	14.8 ± 3.5	124 ± 4.4	135 ± 2.3	68.3 ± 5.0	68.0 ± 3.2
5,000	37.7 ± 5.5	86.3 ± 4.4	146 ± 5.6	157 ± 4.2	15.3 ± 4.9	16.1 ± 3.4	227 ± 4.2	204 ± 3.12	70.4 ± 5.7	72.2 ± 3.4
Positive control	1702 ± 199*	1719 ± 200*	2,882 ± 149*	2,455 ± 749*	2,157 ± 402*	2,311 ± 401*	1,289 ± 135*	1,274 ± 131*	1,323 ± 124*	1,356 ± 134*
Negative control	34.2 ± 2.8	32.12 ± 2.80	134 ± 20	167 ± 19	12.2 ± 2.8	151 ± 2.7	127 ± 22.5	149 ± 21	71.7 ± 13.2	77.8 ± 17.5

Data shown as mean ± SD.

*Statistical significance compared to control, p < 0.05.-S9 positive controls for Strain 98: 2-nitrofluorene (2NF); Strains TA, 97a, TA, 100; TA, 1535: Sodium-azide (NaN3); Strain WP2: methyl-methanesulfonate (MMS).

+S9 (5%) positive controls for Strain 98: Benzo(a)pyrene (BP); Strains TA, 97a, TA, 100; TA, 1535, WP2: 2-aminoanthracene (2AAn).

**TABLE 10:** Results of the bacterial reverse mutation assay with Organic Turkey Tail M2-101-03 powder.

	Revertant colonies per plate									
Concentration (µg/plate)	*Salmonella typhimurium*								*Escherichia coli*	
	TA98		TA100		TA1535		TA97a		WP2 *uvrA*	
	-S9	+S9 (5%)	-S9	+S9 (5%)	-S9	+S9 (5%)	-S9	+S9 (5%)	-S9	+S9 (5%)
50	25.5 ± 2.2	28.3 ± 2.6	128 ± 2.3	165 ± 4.1	11.7 ± 2.5	14.1 ± 3.3	113 ± 2.0	159 ± 2.7	60.3 ± 3.5	66.2 ± 2.2
100	31.2 ± 3.0	35.3 ± 2.4	134 ± 2.2	130 ± 2.2	12.2 ± 2.4	12.8 ± 3.2	123 ± 3.1	145 ± 2.1	66.3 ± 3.5	67.3 ± 3.3
500	32.3 ± 2.3	35.3 ± 4.1	140 ± 2.5	152 ± 2.3	13.0 ± 2.3	15.3 ± 2.4	121 ± 2.6	133 ± 3.5	66.2 ± 2.2	69.0 ± 3.1
1,000	35.3 ± 3.1	37.1 ± 2.1	146 ± 3.6	155 ± 3.5	13.9 ± 3.2	16.0 ± 2.3	125 ± 2.3	135 ± 3.1	69.3 ± 3.0	69.2 ± 3.1
5,000	36.3 ± 3.5	88.3 ± 4.4	145 ± 3.4	161 ± 3.2	16.0 ± 3.9	16.9 ± 2.9	227 ± 3.2	205 ± 2.3	72.6 ± 3.4	70.7 ± 2.6
Positive control	1704 ± 199*	1730 ± 198*	2,888 ± 140*	2,461 ± 746*	2,141 ± 403	231 ± 41*	1,284 ± 124*	1,249 ± 125*	1,315 ± 115*	1,361 ± 124*
Negative control	34.3 ± 2.2	34.2 ± 3.2	133 ± 15	169 ± 14	12.7 ± 2.3	154 ± 2.5	129 ± 13.0	145 ± 13.1	71.0 ± 12.3	79.1 ± 13.3

Data shown as mean ± SD.

*Statistical significance compared to control, p < 0.05.

-S9 positive controls for Strain 98: 2-nitrofluorene (2NF); Strains TA, 97a, TA, 100; TA, 1535: Sodium-azide (NaN3); Strain WP2: methyl-methanesulfonate (MMS).

+S9 (5%) positive controls for Strain 98: Benzo(a)pyrene (BP); Strains TA, 97a, TA, 100; TA, 1535, WP2: 2-aminoanthracene (2AAn).

#### 3.1.4 *In vivo* mammalian cell micronucleus test

All dose groups tested were well tolerated, with no premature mortality noted in either sex of rats that were treated with Organic Lion’s Mane M2-102-10 powder or Organic Turkey Tail M2-101-03 powder. Across all dose levels, the animals showed no clinical indications that were suggestive of treatment-related effects, including the highest tested dosage of 2000 mg/kg body weight, or in satellite groups. Administration of either test item did not result in any statistically significant increases in the frequency of micronuclei or micronucleated polychromatic erythrocytes (PCEs) at 24 or 48 h post-treatment, compared to the vehicle control group (Tables 11, 12, respectively). In contrast, the positive control group exhibited a significant increase (p < 0.05) in micronucleated PCEs, confirming the sensitivity and validity of the assay. Collectively, these results indicate that Organic Lion’s Mane M2-102-10 powder and Organic Turkey Tail M2-101-03 powder did not exhibit clastogenic or aneugenic potential under the conditions of this *in vivo* micronucleus assay.

**TABLE 11:** Result of the *in vivo* micronucleus test in mice with Organic Lion’s Mane M2-102-10 powder.

Treatment group	Dose	Number of PCE with micronuclei per 2000 PCE	PCE/NCE ratio
Vehicle control	10 mL/kg bw, 24 h	1.2 ± 0.8	0.74 ± 0.12
Positive control	50 mg/kg bw, 24 h	54.87 ± 10.4**	0.83 ± 0.11
Organic Lion’s Mane M2-102-10 powder	300 mg/kg bw, 24 h	1.3 ± 0.95	0.87 ± 0.07
	1,000 mg/kg bw, 24 h	1.1 ± 0.99	0.86 ± 0.01
	2000 mg/kg bw, 24 h	1.3 ± 0.95	0.82 ± 0.03
Organic Lion’s Mane M2-102-10 powder satellite	2000 mg/kg bw, 48 h	1.1 ± 0.88	0.74 ± 0.01

Data shown as mean ± SD.

**Statistical significance compared to vehicle control, p < 0.01.

PCE, polychromatic erythrocytes; NCE, normochromatic erythrocytes.

Vehicle control–phosphate buffered saline; Positive Control–cyclophosphamide.

**TABLE 12:** Result of the *in vivo* micronucleus test in mice with Organic Turkey Tail M2-101-03 powder.

Treatment group	Dose	Number of PCE with micronuclei per 2000 PCE	PCE/NCE ratio
Vehicle control	10 mL/kg bw, 24 h	1.0 ± 0.7	0.63 ± 0.14
Positive control	50 mg/kg bw, 24 h	53.2 ± 15.2**	0.61 ± 0.15
Organic Turkey Tail M2-101-03 powder	300 mg/kg bw, 24 h	1.4 ± 0.8	0.62 ± 0.19
	1,000 mg/kg bw, 24 h	1.3 ± 0.9	0.51 ± 0.23
	2000 mg/kg bw, 24 h	1.4 ± 0.8	0.62 ± 0.12
Organic Turkey Tail M2-101-03 powder satellite	2000 mg/kg bw, 48 h	1.9 ± 1.0	0.57 ± 0.16

Data shown as mean ± SD.

**Statistical significance compared to vehicle control, p < 0.01.

PCE: polychromatic erythrocytes; NCE: normochromatic erythrocytes.

Vehicle control–phosphate buffered saline; Positive Control–cyclophosphamide.

## 4 Discussion

The findings of these studies indicate that both Organic Lion’s Mane M2-102-10 and Organic Turkey Tail M2-101-03 powders neither exhibited acute toxicity, nor showed any effects following a single oral dose of 2000 mg/kg bw. Additionally, both powders were non-genotoxic in the bacterial reverse mutation assay (up to 5,000 µg/plate) and in the *in vivo* mouse micronucleus assay (up to 2000 mg/kg bw). The subchronic oral toxicity studies showed some statistically significant findings. However, the changes observed in comparison to the vehicle control group remained within the bounds of normal physiological variation, were not associated with any histopathological alterations and considered not as treatment-related effects.

Statistical significance alone does not confirm that an effect is biologically relevant, treatment-related, or adverse (National Toxicology Program, 2025). Conversely, certain effects may not reach statistical significance, yet a comprehensive evaluation of the data may indicate a dose-response relationship, treatment-related patterns, or adverse outcome. Therefore, established scientific principles and best practices, as outlined in the literature (National Toxicology Program, 2025; Palazzi et al., 2016; Pandiri et al., 2017; World Health Organization, 2015) were applied in the interpretation of observations from the subchronic oral toxicity studies involving Organic Lion’s Mane M2-102-10 and Organic Turkey Tail M2-101-03 powders.

In the subchronic study with Organic Lion’s Mane M2-102-10 powder, statistically significant increases in the adrenal gland weights were observed in female rats administered the highest dose. Spleen weights, both absolute and relative to body weight, were significantly elevated in both sexes at the 1,000 mg/kg bw/day dose. A significant reduction in heart weight was observed in both sexes at the 2000 mg/kg bw/day dose. Liver weights were higher at higher doses in males and females, and ovarian weights were significantly elevated in high-dose females. Despite reaching statistical significance, these changes were within 10% of control values and remained within the range of normal biological variability. In the absence of correlating clinical pathology or histopathological findings, these organ weight changes were not considered adverse (National Toxicology Program (NTP), 2025; World Health Organization (WHO), 2015).

Changes reported in the total bilirubin levels in female rats were considered as non-adverse since it was not indicative of liver damage, given the related enzyme parameters such as AST, ALP and cholesterol did not show a commensurate increase (World Health Organization, 2015) and no histopathological findings were reported. TSH level showed a slight increase in the 2000 mg/kg bw/day dose in male rats in comparison to the control group. The change was small in magnitude and indicative of normal variability. Increase in the glucose level noted in males and females at all doses were <10% compared to controls and therefore considered non-adverse (World Health Organization, 2015).

The reductions in hemoglobin in the 2000 mg/kg bw/day dose group and in total red cell count in the 1,000 mg/kg bw/day dose group in both sexes compared to the controls; a decrease in platelet count noted at the 500 mg/kg bw/day dose in both male and female rats, as well as in female rats at the 2000 mg/kg bw/day dose, were <10% compared with controls. The increase in monocyte count observed in the 2000 mg/kg bw/day dose group in both the male and female rats was well below 3-fold and indicative of normal variability. Based on the stated comparisons relative to control groups, the changes were considered non-adverse as per the criteria by World Health Organization, (2015).

In the subchronic study with Organic Turkey Tail M2-101-03 powder, statistically significant findings included weight changes in adrenals, brain, kidneys, heart and spleen in male and female rats compared to controls. The weight of lungs in male rats showed a small but statistically significant decrease at higher doses. Testes weights (absolute and relative to body weight) at 2000 mg/kg bw/day dose were significantly increased compared with controls in males. The observed changes in organ weights were not interpreted to be adverse, given that they were within normative ranges of physiological variation and lacked changes in clinical markers and tissue morphology (Palazzi et al., 2016; Pandiri et al., 2017; World Health Organization, 2015).

Alanine Amino Transferase (ALT) was significantly increased in female rats at higher doses compared to the control group. However, other markers for liver damage such as total bilirubin, AST, ALP, cholesterol did not show any marked increase, and no histopathological observations of concern were reported. Triglycerides increased significantly in the 2000 mg/kg bw/day dose group in males and females compared to the controls. The thyroid hormones (T3, T4, TSH) also showed a statistically significant increase (p < 0.05) at higher doses in males and females. These observations were within the normal laboratory ranges and considered non-adverse (Palazzi et al., 2016; Pandiri et al., 2017; World Health Organization (WHO), 2015).

A decrease was noted in hemoglobin, total red blood cell count, total WBC, packed cell volume in both male and female rats at higher doses compared to control groups. Mean corpuscular volume (MCV) was significantly increased only in female rats at only the 1,000 mg/kg bw/day dose level compared to the control group. Mean corpuscular hemoglobin concentration (MCHC) decreased at 1,000 mg/kg bw/day and increased at 2000 mg/kg bw/day in both males and females. Neutrophils and eosinophils were significantly lower at higher doses in male rats compared to the control group. A statistically significant reduction in Monocytes was seen only in female rats in only the 2000 mg/kg bw/day dose group compared to the control. The levels of the hematology parameters observed were within the normal ranges; some changes did not demonstrate a dose-response and the changes between treated groups were within the levels that qualify as non-adverse effects (World Health Organization, 2015).

The two products tested in this study were powder preparations of DNA-verified *H. erinaceus* mycelial biomass and fruiting body cultured on whole organic oats (Organic Lion’s Mane M2-102-10 powder) and *T. versicolor* mycelial biomass and primordia cultured on organic whole oats (Organic Turkey Tail M2-101-03 powder). They were manufactured by M2 Ingredients Inc. In the United States using a controlled process in an indoor facility with environmental monitoring. Therefore, the resulting products meet established specifications, and any inherent variability is typical of any natural product.

While there is some published literature on the potential toxicity of *H. erinaceus* and *T. versicolor* preparations, it is mainly on extract preparations, made from select mushroom components. Some preparations were enriched in bioactive compounds (Chen et al., 2022; Lai et al., 2011; Lakshmanan et al., 2016; Li et al., 2014a; Li et al., 2014b). There are no studies in the published literature till date on the toxicity of mushroom powders grown on oats. The composition of *H. erinaceus* and *T. versicolor* powders made by M2 Ingredients may be different in comparison to the extracts, wildcrafted mushrooms, and fungi grown on alternative substrates. Furthermore, the toxicity profile of extracts and powders made from different parts of the mushrooms (e.g., fruiting body, mycelium, or primordia) may be different, and the data from published literature may not be directly applicable to the specific composition of the *H. erinaceus* and *T. versicolor* fermentation preparations cultivated on organic oats. Therefore, the toxicity evaluations carried out on the Organic Lion’s Mane M2-102-10 and Organic Turkey Tail M2-101-03 powders provide essential data supporting their safety under the conditions tested.

## 5 Conclusion

Comprehensive toxicological evaluations—including acute and subchronic oral toxicity studies, as well as *in vitro* and *in vivo* genotoxicity assays—were carried out to support the safety assessment of Organic Lion’s Mane M2-102-10 powder and Organic Turkey Tail M2-101-03 powder. Both substances were found to be non-genotoxic and produced no treatment-related adverse effects at any tested dose, including the highest administered dose, in either acute or 90-day repeated-dose oral toxicity studies. The “no observed adverse effect level” (NOAEL) for both powders was 2000 mg/kg body weight/day, the maximum level tested in this study on male and female rats. These findings align with existing literature demonstrating the low toxicity profiles of *Hericium erinaceus* and *T. versicolor*, and provide additional evidence supporting the safety of these powder preparations up to 2000 mg/kg body weight/day.

## Data Availability

The raw data supporting the conclusions of this article will be made available by the authors, without undue reservation.
